# Real-World Patients’ Diagnosis-to-Treatment Journey with Nontuberculous Mycobacterial Pulmonary Disease: A Cross-Sectional Survey

**DOI:** 10.1007/s40121-024-01015-z

**Published:** 2024-07-10

**Authors:** Kozo Morimoto, Jack R. Gallagher, Dirk Wagner, David E. Griffith, Jakko van Ingen

**Affiliations:** 1 Respiratory Disease Center, Fukujuji Hospital, Japan Anti-Tuberculosis Association, 3-1-24 Matsuyama Kiyose, Tokyo, 204-8522 Japan; 2 Clarity Pharma Research LLC, Spartanburg, SC USA; 3 Division of Infectious Diseases, Department of Medicine II, Freiburg University Medical Center, Faculty of Medicine, University of Freiburg, Freiburg, Germany; 4 Division of Mycobacterial and Respiratory Infections, Department of Medicine, National Jewish Health, Denver, CO USA; 5 Radboudumc Center for Infectious Diseases, Department of Medical Microbiology, Radboud University Medical Center, Nijmegen, The Netherlands

**Keywords:** Culture conversion, Europe, Japan, Nontuberculous mycobacterial pulmonary disease, Real-world, Survey, Treatment

## Abstract

**Introduction:**

The incidence and prevalence of nontuberculous mycobacterial pulmonary disease (NTM-PD) are increasing globally. Approximately 80% of NTM-PD cases in Japan and five countries within Europe (Eur5; France, Germany, Italy, Spain, and the UK) are caused by *Mycobacterium avium* complex (MAC). This study describes the clinical decision-making process associated with the management of patients with NTM-PD in Japan and the Eur5.

**Methods:**

We analyzed data from a survey conducted between July 2013 and October 2013 among physicians treating patients with NTM-PD in clinical practice to compare the healthcare settings, clinical presentation, and patient management in Japan and the Eur5.

**Results:**

Overall, 619 physicians (Japan, 173; Eur5, 446) participated in the survey. Most patients in Japan (85%) and the Eur5 (79%) were diagnosed with MAC-PD. Patients were managed generally in hospital-based outpatient clinics (117/173, 68%) in Japan and research/teaching hospitals affiliated with medical schools (140/446, 31%) in the Eur5. The most common reason for delaying treatment was the patient’s symptoms not being considered serious enough for treatment (55/128, 43%) in Japan and awaiting results of antimicrobial susceptibility testing (44/151, 29%) in the Eur5. Culture negativity was less commonly achieved after treatment in patients in Japan versus those in the Eur5 (31% [73/238] vs. 70% [300/426], *p* < 0.0001). In treatment phases that were either completed or discontinued, the primary goal was symptomatic improvement, followed by achieving culture conversion, in both Japan and the Eur5. Overall, 19% (16/85) of physicians in Japan and 43% (220/511) in the Eur5 were “entirely satisfied” with their patients’ treatment outcomes.

**Conclusions:**

Similarities and differences exist in the healthcare settings, clinical presentation, and management of patients with NTM-PD in Japan and the Eur5. Insufficient consideration of culture status by physicians, delayed treatment initiation, and symptom-based cessation emphasize the need for educational efforts on the guideline-based strategies.

**Supplementary Information:**

The online version contains supplementary material available at 10.1007/s40121-024-01015-z.

## Plain Language Summary

Mycobacteria are microorganisms that cause a disease in the lungs known as nontuberculous mycobacterial pulmonary disease (NTM-PD). The number of people with NTM-PD is increasing globally. This study was a survey of doctors who treated people with NTM-PD in Japan and Europe and aimed to understand geographical similarities and differences in the management, treatment, and health of people with NTM-PD. In the survey, treatment for NTM-PD was found to be often delayed or not started. In Japan, this was most commonly because the individual’s symptoms were not thought to be serious enough and in Europe because of delays in laboratory testing needed to decide which antibiotic treatment should be used. The most common treatment goal in both Japan and Europe was improvement in the individual’s symptoms. Clinical guidelines recommend continuing treatment for at least 12 months after the person with NTM-PD has tested negative for mycobacteria. There were similarities and differences in the healthcare settings, clinical presentation, and management of people with NTM-PD between Japan and Europe. It is important to ensure uniform implementation of the treatment guidelines for NTM-PD in each clinical setting so that people with NTM-PD experience better health outcomes.

**Supplementary Information:**

The online version contains supplementary material available at 10.1007/s40121-024-01015-z.

## Key Summary Points

**Table:** 

The incidence and prevalence of nontuberculous mycobacterial pulmonary disease (NTM-PD) are increasing globally; understanding regional clinical practice patterns is critical to identify gaps in healthcare and develop strategies to improve patient outcomes.
This study aimed to identify and compare regional clinical practice patterns related to diagnosis and treatment, patients’ response to treatment, and physicians’ satisfaction with treatment outcomes to gain insights into factors that may contribute to a poor prognosis in Japan and Europe.
Patients were managed predominantly in hospital-based outpatient clinics in Japan and research/teaching hospitals affiliated with medical schools in Europe; the most common reason for treatment delay in Japan was symptoms not being considered serious enough for treatment and in Europe was awaiting results of antimicrobial susceptibility testing; symptomatic improvement was the most common treatment goal in Japan and Europe.
Similarities and differences exist in the healthcare settings, clinical presentation, and management of patients with NTM-PD in Japan and Europe; ensuring uniform implementation of the treatment guidelines would likely result in better prognosis in patients with NTM-PD.

## Introduction

The incidence and prevalence of nontuberculous mycobacterial pulmonary disease (NTM-PD) are increasing globally, especially NTM-PD caused by *Mycobacterium avium* complex (MAC; MAC-pulmonary disease [MAC-PD]) [1–4]. Higher rates of NTM-PD have been reported in older individuals [5, 6] and people with comorbid chronic obstructive pulmonary disease (COPD) or bronchiectasis [7]. NTM-PD has two primary but often overlapping phenotypes: the fibrocavitary form, which occurs in patients with pre-existing pulmonary diseases, such as COPD, bronchiectasis, previous tuberculosis, or other structural lung disease, and the nodular-bronchiectatic form, which primarily affects the lingula and middle lobe and tends to occur primarily in the middle-aged and elderly female population [4].

We have previously reported results from a cross-sectional survey of physicians in the US, Japan, and Europe [1, 8, 9]. In one of the reports [8], we reported that MAC was the predominant causative agent of NTM-PD in Japan (∼ 85%) and in five European countries (Eur5; France, Germany, Italy, Spain, and the UK; ∼ 79%) [8]. We also found important differences in the epidemiology of NTM-PD and the diagnostic and treatment practices for NTM-PD between Japan and the Eur5 [8, 9]. Most patients with NTM-PD in Japan had mild disease (67%) as assessed by their physicians, whereas most patients in the Eur5 had moderate disease (59%) [8]. Patients in Japan more commonly presented with the nodular-bronchiectatic form than in patients in the Eur5 (∼ 60% vs. 39%) [8]. Among patients with MAC-PD, approximately 42% in Japan and 9% in the Eur5 received the recommended three-drug macrolide-based regimen for > 6 months [10], indicating poor adherence to the 2007 American Thoracic Society/Infection Disease Society of America treatment guidelines in real-world clinical practice [8, 10]. Limited adherence to guidelines in Japan was subsequently confirmed by an analysis of national health insurance data [11]. Thus far, the cross-sectional survey described above is the only reported study that evaluated a nationally representative sample of patients with NTM-PD in Japan and in each Eur5 country [8].

Given the previous findings of poor adherence to guidelines and differences in disease severity, form of NTM-PD, and adherence to guidelines for treating MAC-PD between countries, it is essential to grasp clinical practice patterns related to NTM-PD in more detail to develop strategies to improve patient outcomes [8, 11]. We herein provide novel analyses of data from a previously reported cross-sectional survey [8] and describe in detail the similarities and differences in healthcare settings, clinical presentation of patients, factors influencing initiation of treatment for NTM-PD, choice of oral antibiotic, oral antibiotic prescription patterns, patients’ response to treatment, and physicians’ satisfaction with treatment outcomes.

## Methods

### Study Design, Setting, and Participants

A cross-sectional survey was conducted among physicians from Japan and the Eur5 between July 18, 2013, and October 13, 2013, as previously reported [8]. The first step in physician selection was the identification of active, patient-care physicians in NTM-PD–treating specialities in each country from a master list containing the contact information of physicians in each speciality developed and continually updated by an international physician research supplier (Medefield) and supplemented by information from a comprehensive project by the European Pharmaceutical Market Research Association (EPHMRA) Foundation [12].

From these lists, physicians were selected randomly to participate in a screening survey. To obtain a nationally representative sample of physicians from each country, a multi-layered, country-specific weighting approach, similar to propensity score weighting, was used to adjust for over- or underrepresentation of the sampled population to the expected distribution of the population of patients with NTM-PD in the corresponding country (Supplementary Material) [8]. To be eligible to participate in the survey, physicians were required to be currently managing at least one patient with a confirmed diagnosis of NTM-PD in the prior 12 months and have complete records related to the patient’s NTM-PD [8].

To avoid bias relating to the selection of patients by physicians for the survey (i.e., by disease severity, response to treatment, or other factors), participating physicians were directed to extract anonymized data from medical records for their most recent four patients. Patients had to have a confirmed diagnosis of NTM-PD, could currently be living or deceased, and had to have been seen in the prior 12 months. In a previous publication based on this survey, MAC was reported as the predominant causative agent of NTM-PD (in approximately 80% of cases), followed by *Mycobacterium abcessus* [8]. Therefore, treatment regimens reported in this survey were compared with guideline-based regimens associated with MAC [10].

To facilitate screening, the minimal physician sample size was determined in each of the Eur5 countries by setting the two-sided significance level (type 1 error rate) at 0.05, expected proportion in population at 0.2 and precision at 0.10, which required 62 physicians. The precision for Japan was increased to 0.06, which required a minimum of 171 physicians.

### Study Variables and Outcomes

The healthcare settings, clinical presentation, management, and treatment response of patients with NTM-PD were compared between Japan and the Eur5 (Supplementary Material).

### Statistical Analysis

*Z* tests with Bonferroni correction for multiple comparisons were used for categorical comparisons, and independent-sample *t*-tests were used to compare means of continuous data. For comparison of proportions, *N*−1 chi-squared tests were used. Unless otherwise noted, a *p* value < 0.05 was considered statistically significant. Statistical tests were performed for all comparisons between Japan and the Eur5; *p* values < 0.05 are shown in the text, figures, or tables; *p* values are not shown for non-significant results. Missing data were minimized through programming the electronic questionnaire to prevent skipped answers and allowing the participants to easily save their answers and continue the questionnaire later.

### Ethical Approval

This retrospective, observational study was verified as exempt from an ethical approval by Solutions IRB (Yarnell, AZ, USA) according to the Code of Federal Regulations under 45 CFR 46 section 101 (b)(4), as the study used previously existing, anonymized, and deidentified patient data. For this type of study, informed consent is waived/not applicable.

## Results

### Participants

Of the 3590 physicians contacted (Japan, *n* = 620; Eur5, *n* = 2970), 3154 (88%) agreed to answer the screening questions (Japan, *n* = 569; Eur5, *n* = 2585) to determine whether they were eligible for the study. Among the physicians who were screened, 997 (31%) met the eligibility criteria (Japan, *n* = 240; Eur5, *n* = 757) and 619 (62%) of study-qualified physicians participated in the study (Japan, *n* = 173; Eur5, *n* = 446), thereby exceeding the minimum sample-size requirements in Japan and the Eur5. Anonymized treatment information for a total of 1429 patient records (Japan, *n* = 417; Eur5, *n* = 1012) were obtained from the physicians [8].

### Characteristics and Clinical Presentation of Patients with NTM-PD

Patients in the Eur5 who were included in the study had a mean ± standard deviation (SD) age of 55.7 ± 15.8 years, 37% were female, 25% were never smokers, 33% had COPD, and 22% had bronchiectasis. Patients from Japan had a mean age of 67.0 ± 11.5 years, 65% were female, 65% were never smokers, 13% had COPD, and 25% had bronchiectasis [8].

Patients in Japan experienced symptoms for a mean ± SD of 8.9 weeks before first presenting with NTM-PD to a physician, whereas those in the Eur5 experienced symptoms for a mean of 7.8 weeks. The mean body weight of patients in Japan with NTM-PD was lower than that of patients in the Eur5 (51 vs. 69 kg) (Japan, *n* = 417; Eur5, *n* = 1012) (Table 1). The proportion of underweight patients (body mass index < 18.5 kg/m^2^) was higher in Japan than in the Eur5 (23% [96/417] vs. 6% [61/1012]). The percentage of patients known to be immunocompromised (as reported by the responding physician) at first presentation with NTM-PD was lower in Japan than in the Eur5 (8% [32/417] vs. 32% [321/1012], *p* < 0.0001). Within 12 months prior to the survey, patients with NTM-PD in Japan had a lower incidence of pneumonia (17% [69/417] vs. 30% [299/1012], *p* < 0.0001), acute bronchitis (18% [73/417] vs. 33% [336/1012], *p* < 0.0001), and allergic bronchopulmonary aspergillosis (1% [3/417] vs. 3% [28/1012],* p* = 0.0253) than those in the Eur5. The percentage of patients with NTM-PD without any of these comorbidities was higher in Japan than in the Eur5 (68% [283/417] vs. 42% [421/1012], *p* < 0.0001). The use of concomitant medications such as β_2_-agonists (1% [2/179] vs. 19% [128/690]), inhaled combination steroids (1% [2/179] vs. 16% [109/690]), oral steroids (3% [5/179] vs. 14% [96/690]), and anti-reflux therapy (1% [1/179] vs. 17% [116/690]) was lower in Japan than in the Eur5 (Japan, *n* = 179; Eur5, *n* = 690) (Table 1).

**Table 1:** Characteristics of patients with NTM-PD in Japan and the Eur5

Parameters	Japan (*n* = 417) (%)	Eur5 (*n* = 1012) (%)	*p* value
Sex			
Female	65	27	< 0.0001
Male	35	73	< 0.0001
Mean age, years [8]	67	56	0.0001
Mean body weight, kg	51	69	< 0.0001
BMI category			
< 18.5 kg/m^2^	23	6	< 0.0001
18.5–24.9 kg/m^2^	72	58	< 0.0001
25.0–29.9 kg/m^2^	4	30	< 0.0001
≥ 30 kg/m^2^	0	7	< 0.0001
Diagnostic test results			
HCRT showed multi-focal bronchiectasis with multiple small nodules	60	39	< 0.0001
Nodular or cavitary opacities on chest radiograph	47	41	0.0372
Respiratory diseases			
Asthma	7	17	< 0.0001
Pulmonary fibrosis	2	3	0.2899
Other underlying diseases			
Diabetes mellitus	13	21	0.0004
HIV/AIDS	0	14	< 0.0001
GERD	6	11	0.0035
Renal failure	3	6	0.0194
Rheumatoid arthritis	4	4	1.0000
Congestive heart failure	2	7	0.0002
Benign/malignant neoplasm of the lung, bronchus, or pleura	5	5	1.0000
Other neoplasm	1	2	0.1849
Scoliosis	1	3	0.0253
Cystic fibrosis	0	2	0.0036
Concomitant medications^a^			
β_2_-Agonist	1	19	< 0.0001
Inhaled combination steroid	1	16	< 0.0001
Oral steroid	3	14	< 0.0001
Anti-reflux therapy	1	17	< 0.0001

*BMI* body mass index, *COPD* chronic obstructive pulmonary disease, *Eur5* five countries within Europe that were included (France, Germany, Italy, Spain, and the UK), *GERD* gastroesophageal reflux disease, *HRCT* high-resolution computed tomography, *NTM-PD* nontuberculous mycobacterial pulmonary disease

^a^Japan, *n* = 179; Eur5, *n* = 690

### Healthcare Settings for the Management of Patients with NTM-PD

**Fig. 1:**
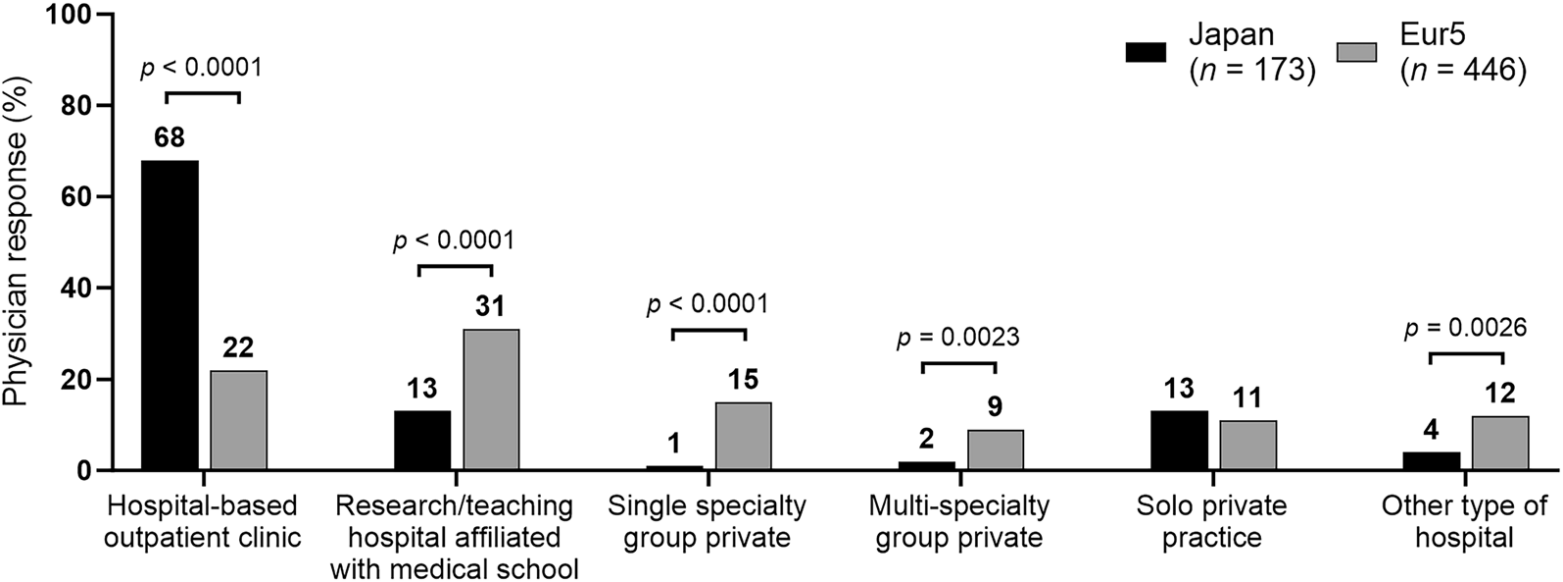
Healthcare settings where patients with NTM-PD were managed in Japan and the Eur5. *Eur5* five countries within Europe (France, Germany, Italy, Spain, and the UK), *NTM-PD* nontuberculous mycobacterial pulmonary disease. Comparisons with no *p* value shown were not significant

The practice setting where patients with NTM-PD were managed in Japan and the Eur5 are summarized in Fig. 1. A higher percentage of physicians in Japan practiced in NTM-PD speciality centers than in the Eur5 (41% [71/173] vs. 22% [98/446], *p* < 0.0001). The length of patient care with the physician treating their NTM-PD ranged from < 1 week to ≥ 71 weeks and was generally longer in Japan than in the Eur5 (< 1 week, 1% [3/417] vs. 0% [4/1012] [*p* = 0.0014]; 1–15 weeks, 15% [61/417] vs. 23% [237/1012] [*p* = 0.0007]; 16–35 weeks, 23% [94/417] vs. 34% [345/1012] [*p* < 0.0001]; 36–70 weeks, 24% [101/417] vs. 18% [184/1012] [*p* = 0.0096]; ≥ 71 weeks, 38% [159/417] vs. 24% [243/1012] [*p* < 0.0001]). Over the previous 12 months, physicians in Japan assessed their patients twice as often as physicians in the Eur5 (mean, 10 vs. 5 visits, *p* < 0.0001).

### Management of Patients with NTM-PD

The percentage of patients who ever received treatment for NTM-PD was 43% (47/110) in Japan and 68% (47/171) in the Eur5 [8]. The most common reasons for not treating patients with NTM-PD in Japan were patients’ symptoms not being considered serious enough for treatment (48%; 53/110), good overall patient health (43%; 47/110), and risk of treatment being considered greater than the expected benefit (38%; 42/110). The most common reasons for not treating patients with NTM-PD in the Eur5 were good overall patient health (30%; 52/171), patients’ symptoms not being considered serious enough for treatment (27%; 47/171), and patients’ refusal to receive treatment for NTM-PD (24%; 41/171) (Fig. S1).

**Fig. 2:**
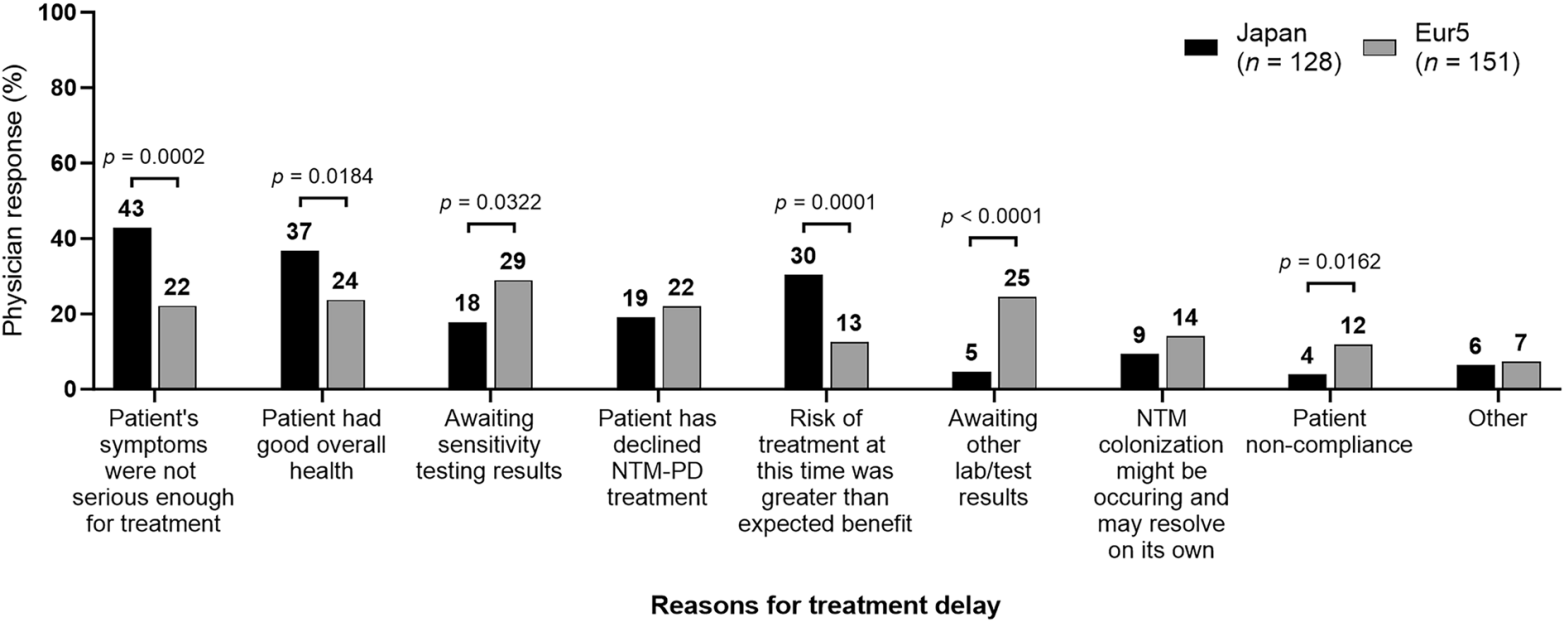
Reasons for treatment delay in patients with NTM-PD in Japan and the Eur5. *Eur5* five countries within Europe (France, Germany, Italy, Spain, and the UK), *NTM-PD* nontuberculous mycobacterial pulmonary disease. Comparisons with no *p* value shown were not significant. Multiple responses; sums may exceed “*n*” or 100%

Treatment was planned in the future for 54% (128/238) of patients in Japan and 47% (151/322) of patients in the Eur5. For these patients, the most common reasons for delaying therapy in Japan were patients’ symptoms not being considered serious enough for treatment (43%; 55/128), good overall patient health (37%; 47/128), and risk of treatment being considered greater than the expected benefit (30%; 39/128). The most common reasons for delaying therapy in the Eur5 were awaiting results of antimicrobial susceptibility testing (29%; 44/151), awaiting other laboratory/test results (25%; 37/151), and good overall patient health (24%; 36/151) (Fig. 2).

**Fig. 3:**
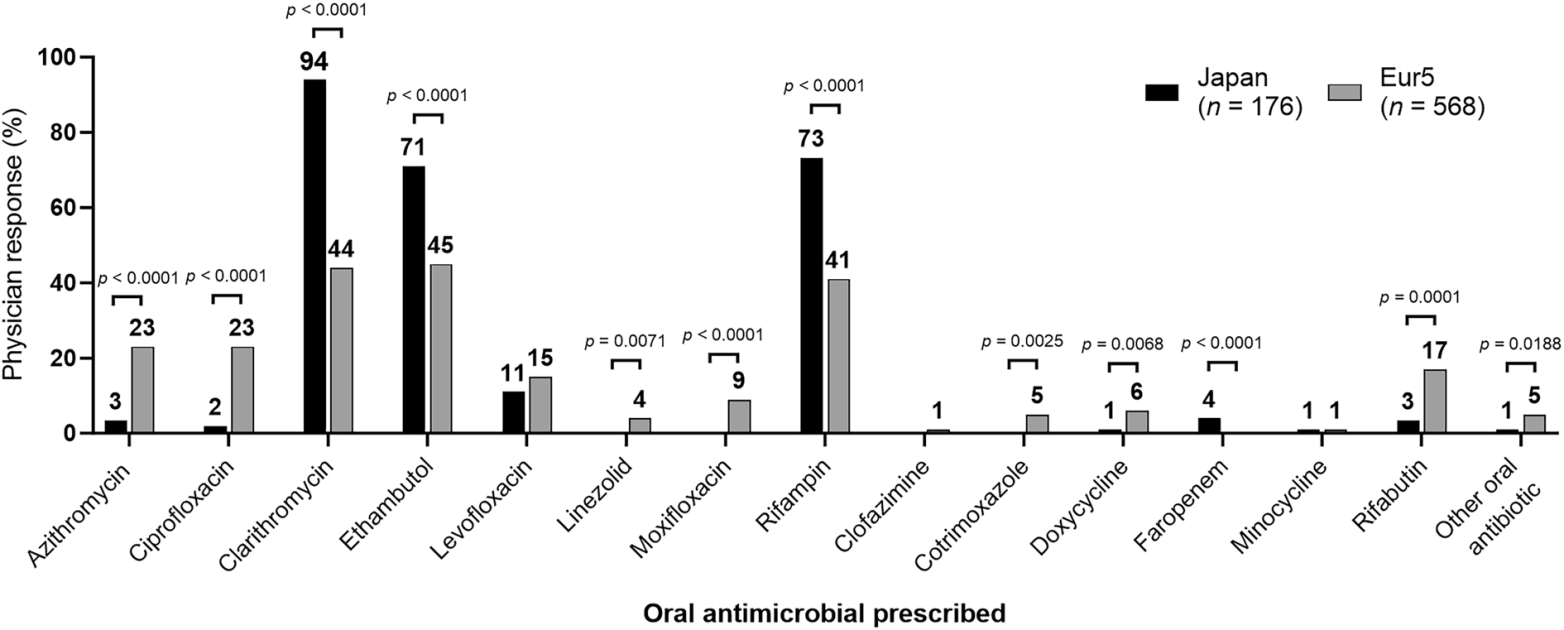
Prescription patterns of oral antibiotics for patients with NTM-PD in Japan and the Eur5. *Eur5* five countries within Europe (France, Germany, Italy, Spain, and the UK), *NTM-PD* nontuberculous mycobacterial pulmonary disease. Comparisons with no *p* value shown were not significant. Multiple responses; sums may exceed “*n*” or 100%

The percentage of patients diagnosed with MAC-PD was 85% in Japan and 79% in the Eur5 [8]. Consistent with the three-drug regimen recommended for MAC-PD in the guidelines [10], clarithromycin, azithromycin, ethambutol, and rifampin were each prescribed to 56% (416/744), 19% (138/744), 51% (378/744), and 49% (364/744) of patients overall, respectively. There was heterogeneity in the pattern of oral antibiotics prescribed for the treatment of NTM-PD in Japan and the Eur5 (Fig. 3). For macrolides, a larger proportion of patients was prescribed clarithromycin in Japan than in the Eur5 (94% [165/176] vs. 44% [251/568]), and a lower proportion was prescribed azithromycin (3% [6/176] vs. 23% [132/568]). Use of rifampin was greater in Japan than in the Eur5 (73% [129/176] vs. 41% [235/568]), and use of rifabutin was lower in Japan than in the Eur5 (3% [6/176] vs. 17% [94/568]). A higher proportion of patients was prescribed ethambutol in Japan than in the Eur5 (70% [124/176] vs. 45% [253/568]). The proportion of patients prescribed fluroquinolones was lower in Japan than in the Eur5 (levofloxacin, 11% [19/176] vs. 15% [87/568]; ciprofloxacin, 2% [4/176] vs. 23% [128/568]; moxifloxacin, 0% [0/176] vs. 9% [52/568]) (Fig. 3). Two of the most common factors affecting the choice of oral antibiotics in Japan were good efficacy (63%; 111/176) and consistency with American Thoracic Society guidelines (51%; 90/176), whereas in the Eur5, the most common important factors were good efficacy (78%; 441/568) and good side effect profile (38%; 214/568) (Fig. S2).

### Monitoring and Response to NTM-PD Treatment

**Fig. 4:**
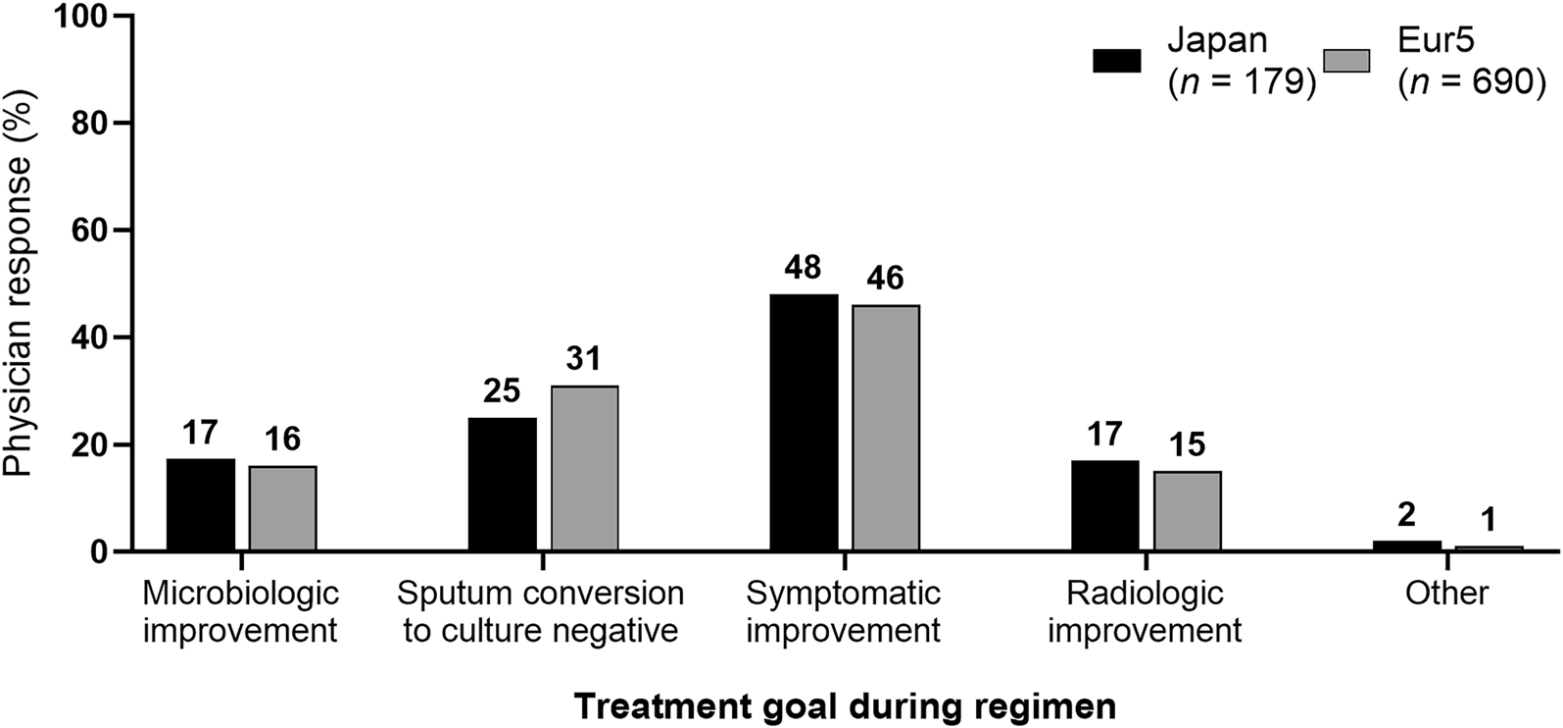
Treatment goal during regimen for patients with NTM-PD in Japan and the Eur5. *Eur5* five countries within Europe (France, Germany, Italy, Spain, and the UK), *NTM-PD* nontuberculous mycobacterial pulmonary disease. Multiple responses gathered over various treatment rounds; sums may exceed “*n*” or 100%. Comparisons with no *p* value shown were not significant. van Ingen et al. [8]

In treatment rounds that were completed or stopped, symptomatic improvement was the most common treatment goal during the treatment round in Japan and the Eur5, followed by culture conversion (Fig. 4) [8]. At the time that the patient chart was examined, sputum cultures for patients post diagnosis who had not yet started treatment had been completed in 74% (176/238) and 49% (158/322) of patients in Japan and the Eur5, respectively. There were differences between Japan and the Eur5 in terms of the percentage of patients with positive culture (77% [183/238] vs. 34% [144/426]), negative culture indicating culture conversion (31% [73/238] vs. 70% [300/426]), and results not yet received since the time of diagnosis and/or following treatment completion (7% [18/238] vs. 15% [65/426]) (Fig. S3).

**Fig. 5:**
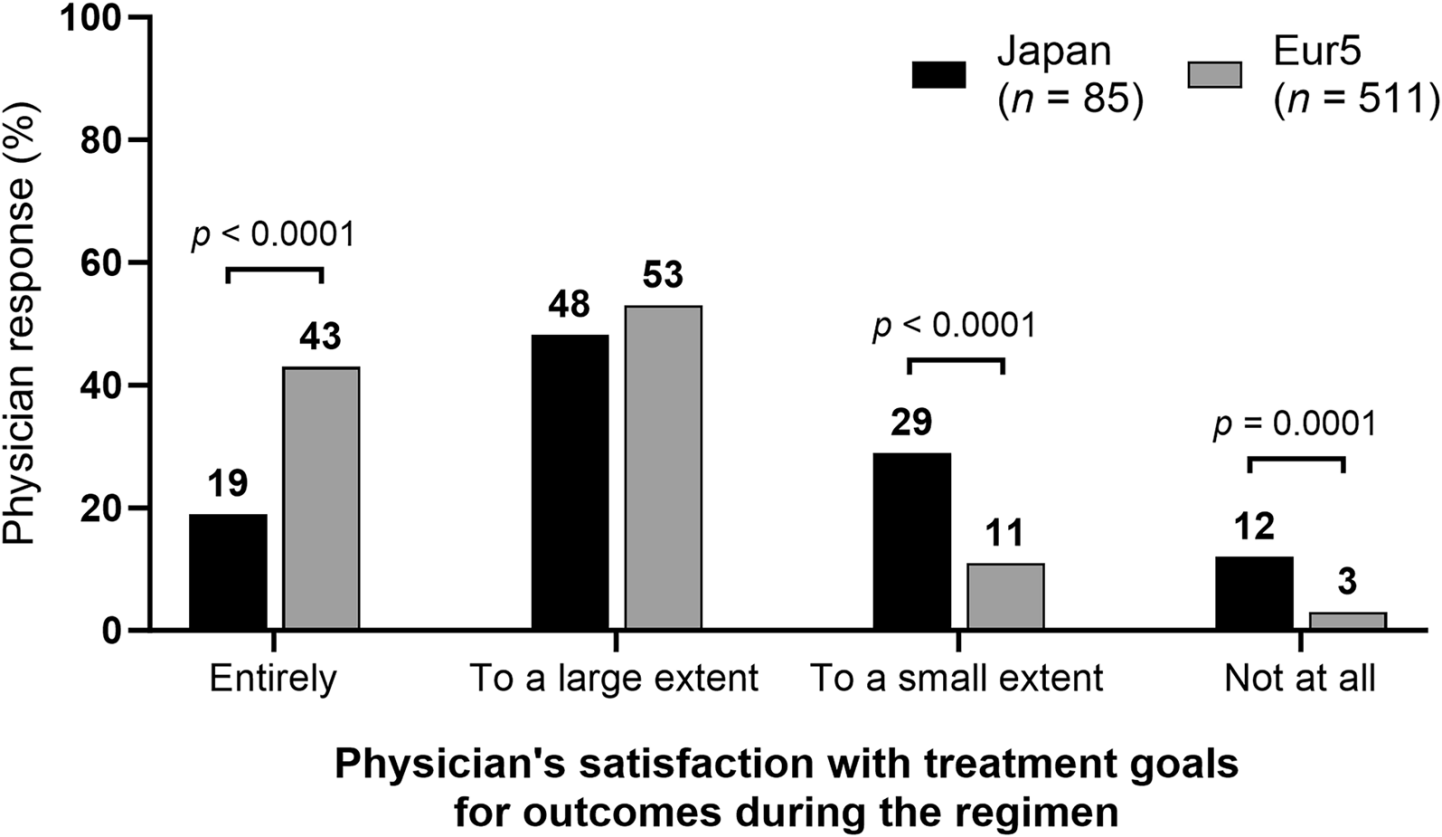
Physicians’ satisfaction with treatment goals during the regimen for patients with NTM-PD in Japan and the Eur5. *Eur5* five countries within Europe (France, Germany, Italy, Spain, and the UK), *NTM-PD* nontuberculous mycobacterial pulmonary disease. Multiple treatment regimens: sums may exceed “*n*” or 100%. Comparisons with no *p* value shown were not significant

The percentage of patients who “greatly improved” based on the most recent radiologic test in Japan was approximately half of that in the Eur5 (19% [78/417] vs. 38% [380/1012]). There were differences in the percentages of patients “not improving,” “improving somewhat,” “greatly improved,” “eradicated,” and “no change” between Japan and the Eur5 (Fig. S4). Overall, 31% [67/215] of patients in Japan and 17% [56/334] in the Eur5 were refractory to treatment (*p* < 0.0001). For patients where NTM-PD treatment had been completed or stopped, a lower percentage of physicians in Japan were “entirely” satisfied with the extent to which treatment goals for outcomes were met during the current treatment regimen than that of physicians in the Eur5 (19% [16/85] vs. 43% [220/511]). Except for the percentage of physicians who were satisfied “to a large extent” (48% [41/85] vs. 53% [272/511]), there were also differences between Japan and the Eur5 in the percentage of physicians who were satisfied “to a small extent” (29% [25/85] vs. 11% [56/511]) and “not at all” (12% [10/85] vs. 3% [13/511]) (Fig. 5).

## Discussion

The results of this study present the similarities and differences in the healthcare settings, clinical presentation, treatment practices, and treatment response of patients with NTM-PD in Japan and the Eur5. Furthermore, the findings bring to light the treatment practices that may result in poor prognosis for patients with NTM-PD in Japan and the Eur5, such as delay in treatment initiation, lack of treatment, prescription patterns of antibiotics, reliance on symptomatic improvement as a criterion for stopping treatment, and the potential lack of adequate consideration of culture conversion status. A lower percentage of physicians in Japan were “entirely satisfied” with their patients’ treatment outcomes than physicians in the Eur5. These insights can help in re-evaluating and improving the diagnostic and management strategies for NTM-PD used by physicians in Japan and the Eur5.

Cultural differences between Japan and the Eur5 could influence clinical patterns and the approach to disease management, which could impact treatment outcomes. Patients with NTM-PD were predominantly treated in NTM-PD speciality centers in a community setting in Japan compared with those treated in an academic setting in the Eur5, which may lead to longer and more frequent physician assessment. This might be caused by the historical background of the medical system in these countries. For example, in Japan, national hospital organizations based on tuberculosis sanatorium and treatment centers have contributed substantially to the care of patients with respiratory diseases, including NTM-PD; prior to the 1990s, most clinical studies and epidemiologic analyses of NTM-PD were conducted by national hospital organizations [13, 14]. Because of the increasing prevalence of NTM-PD, academic hospitals have also been treating and studying this respiratory disease in recent years. Furthermore, annual health check-ups, which are common in Japan, could play a role in the early diagnosis of mild cases and treatment in a community setting [15]. By contrast, more severe cases observed in the Eur5 were treated in an academic setting. Treatment practices for patients with NTM-PD could differ between speciality, academic and non-speciality centers; therefore, the management gaps between the providers in these settings should be assessed. The lower prescription rate of concomitant medications such as β_2_-agonists, inhaled combination steroids, oral steroids, and anti-reflux therapy in Japan than in the Eur5 may be because asthma and gastroesophageal reflux disease were reported in a lower percentage of patients with NTM-PD in Japan than in the Eur5 (7% vs. 17% and 6% vs. 11%, respectively). There is a need for an integrated NTM-PD management beyond antimicrobial therapy, which includes managing comorbidities and implementing a personalized pulmonary rehabilitation plan and airway clearance techniques to improve symptoms, exercise capacity, and health-related quality of life [16]. Moreover, a multi-disciplinary approach would further improve the management of NTM-PD in addition to that achieved using drug therapy [17].

A limitation to this study is that the survey did not collect data in a way that confirms if patients were prescribed a guideline recommended three-drug regimen, which makes it difficult to compare physicians’ adherence to guideline-based prescribing. In a population-based cross-sectional study conducted in Japan, 30.8% of patients received standard treatment [e.g., (1) macrolide, rifamycin, and ethambutol; (2) macrolide and ethambutol], 37.3% received non-standard treatment [e.g., (1) only macrolides, (2) macrolides and rifamycin, or (3) macrolides and fluoroquinolone], and 31.9% received no treatment [18]. In this study clarithromycin was prescribed more commonly than azithromycin in both the Eur5 and Japan. Although this is not consistent with the 2020 guidelines for NTM-PD, which recommend azithromycin over clarithromycin as part of the multidrug regimen for treatment in macrolide-susceptible MAC-PD [19], at the time the study was conducted in 2013 the guidelines available (from 2007) did not state a preference [10]. In addition, clarithromycin was the only guideline that recommended macrolide with an indication that was covered by Japan’s national insurance program in 2013. With 85% of NTM-PD cases in Japan reported to be MAC-PD, this could explain the lower use of azithromycin in Japan versus the Eur5 in this study.

Although the study was conducted before the 2020 guidelines for NTM-PD were published [19], the survey results revealed that there were deviations from the 2007 recommendation [10] in real-world clinical practice in Japan and the Eur5. Patients with good overall health, mild symptoms, and awaiting results for antimicrobial susceptibility testing and other laboratory tests were the main reasons for the lack of or delay in therapy initiation in Japan and the Eur5 in this study. In our previous publication, the variables influencing the treatment decision in patients with NTM-PD in Japan and the Eur5 were determined [8]. Patients assessed by their physician to have moderate or severe symptoms at presentation and those aged > 60 years, living in Spain and Italy, having sputum specimens for acid-fast bacilli analysis being used for diagnosis, having fever as a symptom at the first presentation, and having poor/very poor or fair physician-based rating of overall health were also more likely to be treated [8]. The smaller proportion of patients starting treatment in Japan versus the Eur5 is notable and may be due to differences in disease severity between the two populations. As reported previously, in this survey most patients in Japan were considered by their physicians to have mild disease, whereas most patients in the Eur5 had moderate or severe disease [8]. At the same time, patients in Japan may have been perceived to have less severe disease and, as a result, experienced a delay in treatment initiation, which is understood as a watchful waiting strategy. The perception by Japanese doctors was that the more limited radiographic improvement and smaller proportion of patients experiencing culture conversion might be due to deviation from the standard treatment and frequent recurrences, which have not been noticed in clinical practices. A significantly larger proportion of patients in the Eur5 were prescribed linezolid and moxifloxacin compared with Japan. In European and US guidelines, clofazimine, moxifloxacin, and linezolid have been described as regimen options for resistant refractory NTM-PD with cavitation. In Japan, these antibiotics are not reimbursed by insurance, and the 2012 Japanese NTM-PD treatment guidelines did not include regimen options for such cases, which might explain the discrepancies in the use of these antibiotics between Japan and the Eur5.

Symptom-based NTM-PD management could lead to more advanced diseases, such as cavitary lesions, severe bronchiectasis, and increased bacterial load (as measured by time-to-positivity of the mycobacteria growth indicator tube automated broth culture system) [20], which are associated with poorer outcomes and more cases of refractory disease. Therefore, regular radiology and sputum examinations are warranted for the decision of initiating treatment, provided that these risk factors for refractory outcomes are not already present at the first diagnosis, leading to immediate treatment recommendation and consideration of treatment intensification [19, 21]. Moreover, symptomatic improvement was the most common treatment goal. The optimal treatment duration, as defined by current guidelines published after these data were collected, for NTM-PD is defined as ≥ 12 consecutive months during which culture negativity is maintained [19]. A paucity of studies and differences in patient characteristics, practice settings, and resources hinder the establishment of an optimal treatment duration for NTM-PD [19]; however, two studies have indicated that the treatment duration could change based on disease severity as determined by radiologic evaluation [22, 23]. Nevertheless, symptomatic worsening is associated with treatment initiation and poorer health-related quality of life; for patients who achieve microbiologic conversion, health-related quality of life and survival are improved, highlighting the importance of culture conversion as a treatment goal [24, 25]. Educational initiatives to increase guideline-based therapy, particularly regarding culture conversion, could improve the treatment success rate and lower the relapse rate, ultimately leading to reduced patient mortality.

Overall, the percentage of patients in Japan who had a “greatly improved” status on the most recent radiologic test was half of that in the Eur5. However, patients in Japan may have had less potential to show great improvement as they were generally diagnosed at an earlier disease stage than those in the Eur5. Poor prognosis on radiologic examination and a higher number of refractory patients in Japan than in the Eur5 may be the reasons for a lower percentage of physicians in Japan being “entirely” satisfied with the treatment outcomes than that in the Eur5 (19% vs. 43%). The high rate of refractory NTM-PD in Japan may be due to the use of a substandard treatment regimen, including a shorter than recommended duration of treatment (59% of patients received treatment for > 6 months and 41% received treatment for > 1 year) and the administration of a macrolide without ethambutol or rifampicin [11]. Of note, the nodular-bronchiectatic form is more common in Japan than in the Eur5 [8], which may result in increased rates of reinfection in patients in Japan during and after treatment, similar to that reported in the US [26]. Knowledge sharing and increasing the awareness of guideline recommendations regarding the management of NTM-PD, as well as a systematic treatment approach, are essential for improving the current situation.

An important limitation of this study is that the survey was completed before the release of the latest guideline recommendations in 2020; hence, the analysis was based on the 2007 guideline recommendations for NTM-PD [10]. However, this being the only study of its kind and size conducted in Japan and the Eur5 to date, the findings are relevant even today. Adherence to guidelines in Japan and the Eur5 was assessed based on the prescription data of individual antimicrobials instead of their drug class (e.g., macrolides) or the three-drug regimen for MAC-PD; however, the percentage of patients receiving the individual drugs could serve as an accurate reflection of adherence. Another limitation of the survey is the lack of data on antimicrobial sensitivity, which may have provided further insights into the differences in prescribing patterns between Japan and the Eur5. As patients in the Eur5 had a higher prevalence of asthma, it is possible that respiratory symptoms due to underlying airway disease could be mistaken for symptoms of NTM-PD. Other limitations include potential self-report bias because physicians or their designated personnel retrieved and input the survey response data themselves into an online survey instrument and a lack of uniform susceptibility testing as well as insights into the varying patterns of antibiotic prescriptions across diverse populations.

## Conclusions

To our knowledge, this is the only study to include a nationally representative sample of physicians treating patients with NTM-PD in Japan and each Eur5 country and of each physician’s corresponding patients with NTM-PD and highlights the differences in the healthcare settings, clinical presentation, and management of patients with NTM-PD in Japan and the Eur5. It also revealed the inadequacies in the management of patients with NTM-PD in Japan and the Eur5. Insufficient consideration of culture status by physicians and the delay in treatment owing to the lack of severe symptoms emphasize the need for awareness of guideline-based strategies. Ensuring uniform implementation of the treatment guidelines in each clinical setting would result in better prognosis in patients with NTM-PD.

### Supplementary Information

Below is the link to the electronic supplementary material.

Supplementary file1 (PDF 506 KB)

## Data Availability

All data generated or analyzed during this study are included in this published article and its supplementary information files.
