# Supplementary material for: Real-World Patients’ Diagnosis-to-Treatment Journey with Nontuberculous Mycobacterial Pulmonary Disease: A Cross-Sectional Survey
Source: Infect Dis Ther. 2024 Jul 10;13(8):1907–20. doi: 10.1007/s40121-024-01015-z (PMC11266326; doi:10.1007/s40121-024-01015-z)
Supplement: Supplementary file 1 — Supplementary file1 (PDF 506 KB) [file 40121_2024_1015_MOESM1_ESM.pdf]

SUPPLEMENTARY MATERIAL

Real-World Patients' Diagnosis-to-Treatment Journey with Nontuberculous Mycobacterial Pulmonary Disease: A Cross-Sectional Survey

Kozo Morimoto · Jack R. Gallagher · Dirk Wagner · David E. Griffith · Jakko van Ingen

Corresponding author: K. Morimoto

Fukujuji Hospital, Japan Anti-Tuberculosis Association, 3-1-24 Matsuyama Kiyose, Tokyo 204-8522, Japan

e-mail: morimotok@fukujuji.org

J. R. Gallagher

Clarity Pharma Research LLC, Spartanburg, SC, USA

D. Wagner

Division of Infectious Diseases, Department of Medicine II, Freiburg University Medical Center, Faculty of Medicine, University of Freiburg, Freiburg, Germany

D. E. Griffith

Division of Mycobacterial and Respiratory Infections, Department of Medicine, National Jewish Health, Denver, CO, USA

J. van Ingen

Radboudumc Center for Infectious Diseases, Department of Medical Microbiology, Radboud University Medical Center, Nijmegen, The Netherlands

METHODS

Variables

Healthcare Infrastructure for the Management of Patients with NTM-PD

The clinical setting for the management of patients with nontuberculous mycobacterial pulmonary disease (NTM-PD), including hospital-based outpatient clinic, research/teaching hospital affiliated with a medical school, single-speciality group private practice, multi-speciality group private practice, solo private practice and other type of hospitals; proportion of physicians practicing in NTM-PD–treating speciality centers; duration of patient-physician relationship and frequency of patient assessment were compared between Japan and the five countries of the European Union (Eur5; France, Germany, Italy, Spain, and the United Kingdom).

Clinical Presentation of Patients with NTM-PD in Japan and the Eur5

The duration of symptoms before the first presentation, body weight, proportion of underweight patients, comorbidities, concomitant medications, immune status, presentation of NTM-PD on high-resolution chest tomography, number of emergency room visits in the past year, and acute conditions in the past year were compared between Japan and the Eur5.

Management of Patients with NTM-PD

Time since NTM-PD diagnosis to treatment, causes for delay in therapy, causes for lack of therapy, factors affecting the choice of oral antibiotic, cultures after diagnosis in untreated and treated patients, treatment goal during regimen, culture conversion status after

treatment, patients' response to treatment, number of refractory patients and physicians' satisfaction with treatment goals were compared between Japan and the Eur5.

Potential Sources of Bias

The most important potential source of bias relevant to this manuscript were over- or underrepresentation in the sample of various physician and/or patient segments (sampling bias). For a study to accurately represent its target population, each potentially qualified patient's probability of study selection must be known and accurately represented in study findings. These requirements were met by identifying the population size of the active, patient-care physicians in NTM-PD–treating specialities in each country using as a guide the results of a comprehensive project by the European Pharmaceutical Market Research Association Foundation [12] and master lists in each country containing contact information of physicians in each speciality, which are continually updated by an international physician research supplier (Medefield), supplemented by physician's contact information obtained from previous market research studies.

A multi-layered, country-specific weighting approach, similar to propensity score weighting, was used to adjust for over- or underrepresentation of the sampled population to the expected distribution of the corresponding country population of patients with NTM-PD. The factors influencing a patient's survey weight were speciality of the treating physician, patient volume based on physicians' self-estimates of NTM-PD–treated volume and our estimates of country-specific NTM-PD prevalence.

Handling of Quantitative Variables

All study variables were quantitative. Comparisons of Japan and the Eur5 on continuous- and nominal-scale variables were analyzed with appropriate parametric and non-parametric tests, respectively.

Fig. S1 Factors associated with lack of therapy in patients with NTM-PD in Japan and the Eur5

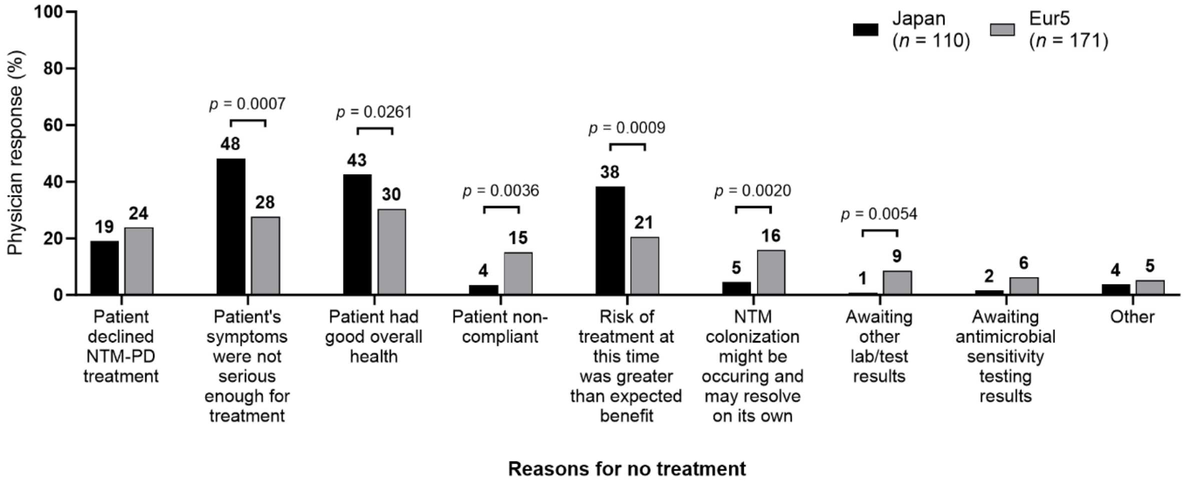

Eur5 5 countries of the European Union (France, Germany, Italy, Spain, and the United Kingdom), *NTM-PD* nontuberculous mycobacterial pulmonary disease

Multiple responses; sums may exceed “n” or 100%

Comparisons with no *p* value shown were not significant

Fig. S2 Factors affecting the choice of oral antibiotics in patients with NTM-PD in Japan and the Eur5

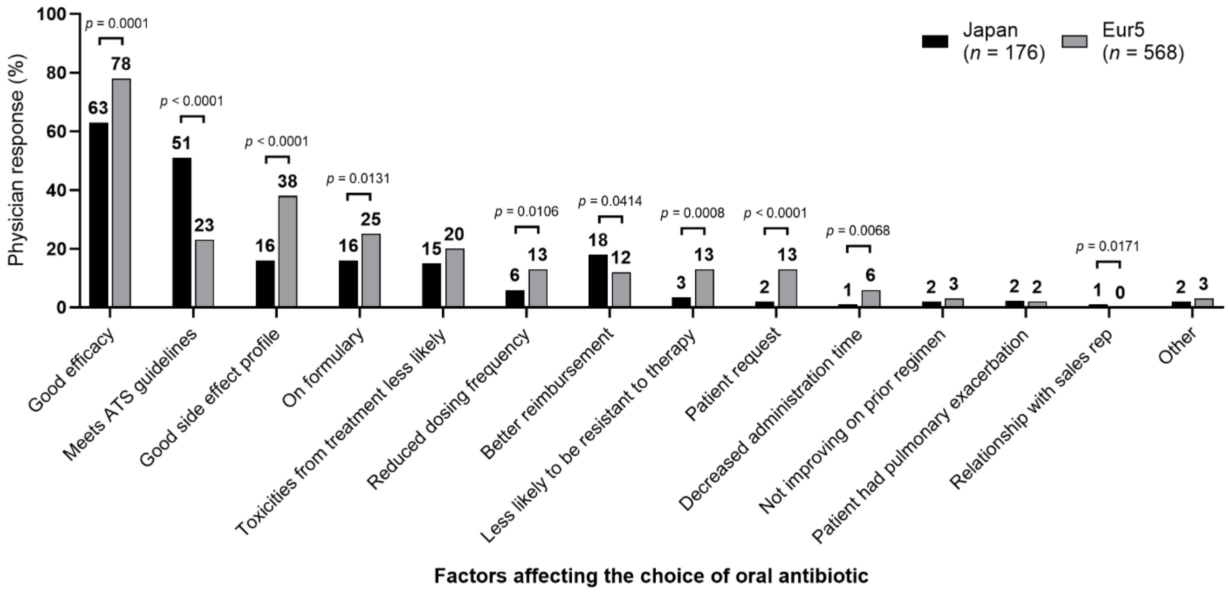

ATS American Thoracic Society, *Eur5* five countries of the European Union (France, Germany, Italy, Spain, and the United Kingdom), *NTM-PD* nontuberculous mycobacterial pulmonary disease, *rep* representative

Multiple responses; sums may exceed “n” or 100%

Comparisons with no *p* value shown were not significant

Fig. S3 Culture conversion status after diagnosis in treated patients with NTM-PD in Japan and the Eur5

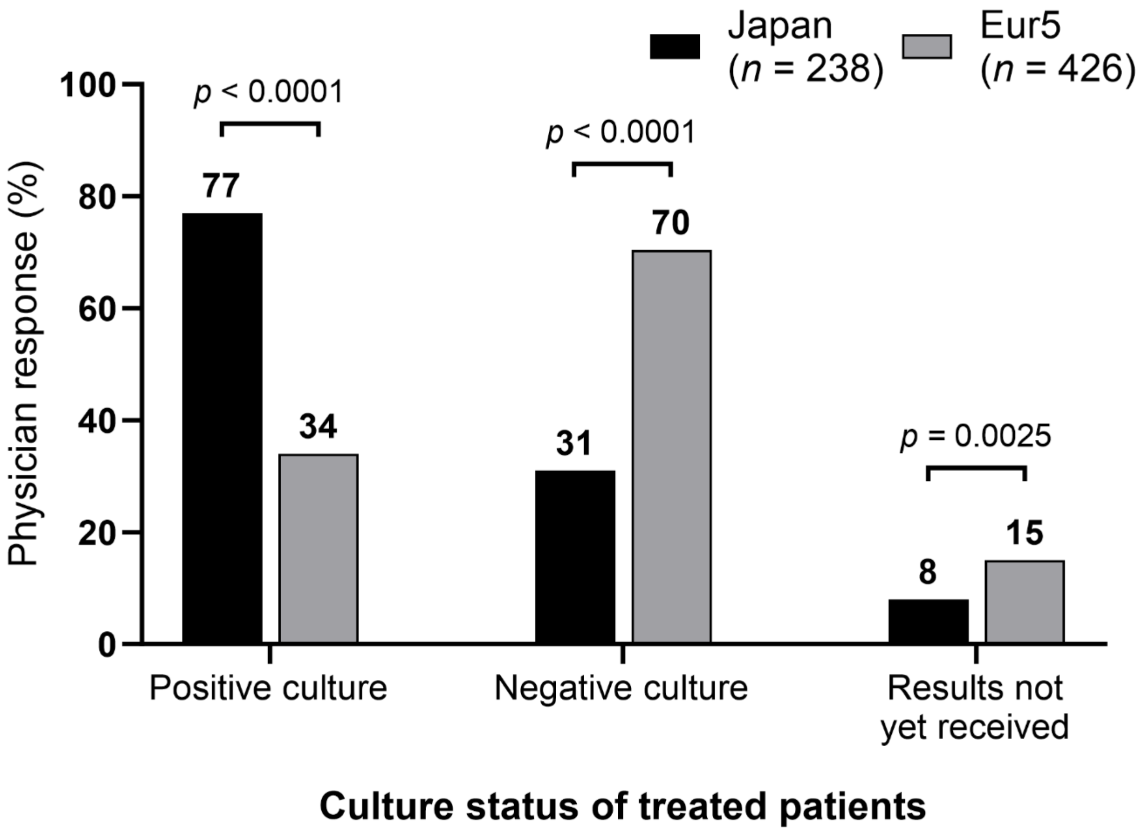

Eur5 5 countries of the European Union (France, Germany, Italy, Spain, and the United Kingdom), *NTM-PD* nontuberculous mycobacterial pulmonary disease.

Positive culture indicates patients have not achieved culture conversion following treatment; negative culture indicates patients have achieved culture conversion following treatment.

Fig. S4 Condition of patients with NTM-PD based on the most recent radiologic test in Japan and the Eur5

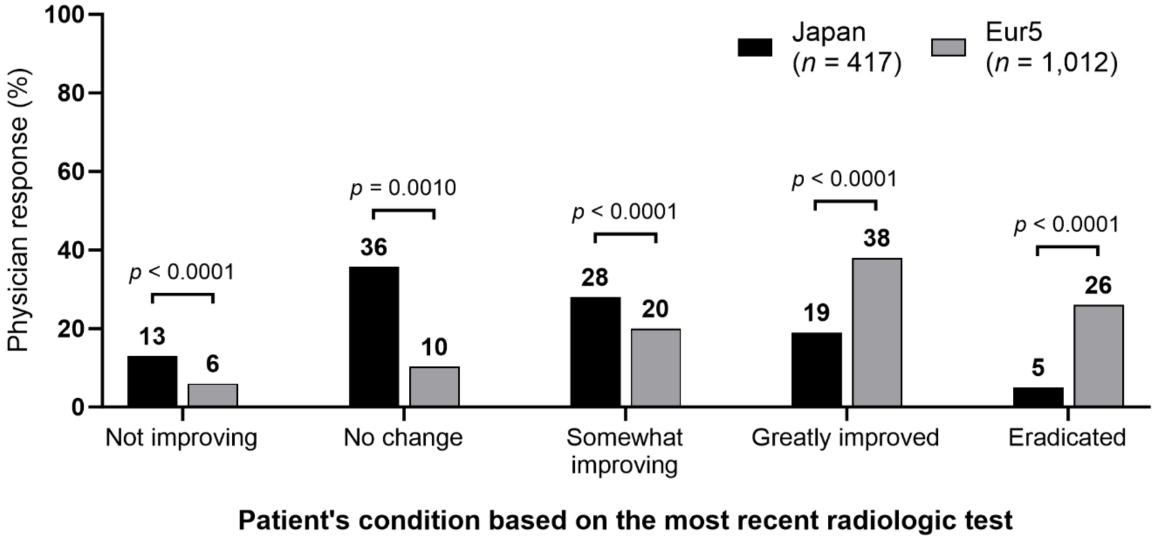

Eur5 5 countries of the European Union (France, Germany, Italy, Spain, and the United Kingdom), *NTM-PD* nontuberculous mycobacterial pulmonary disease
